# Supplementary figures and images for: Potent, specific MEPicides for treatment of zoonotic staphylococci
Source: PLoS Pathog. 2020 Jun 4;16(6):e1007806. doi: 10.1371/journal.ppat.1007806 (PMC7297381; doi:10.1371/journal.ppat.1007806)

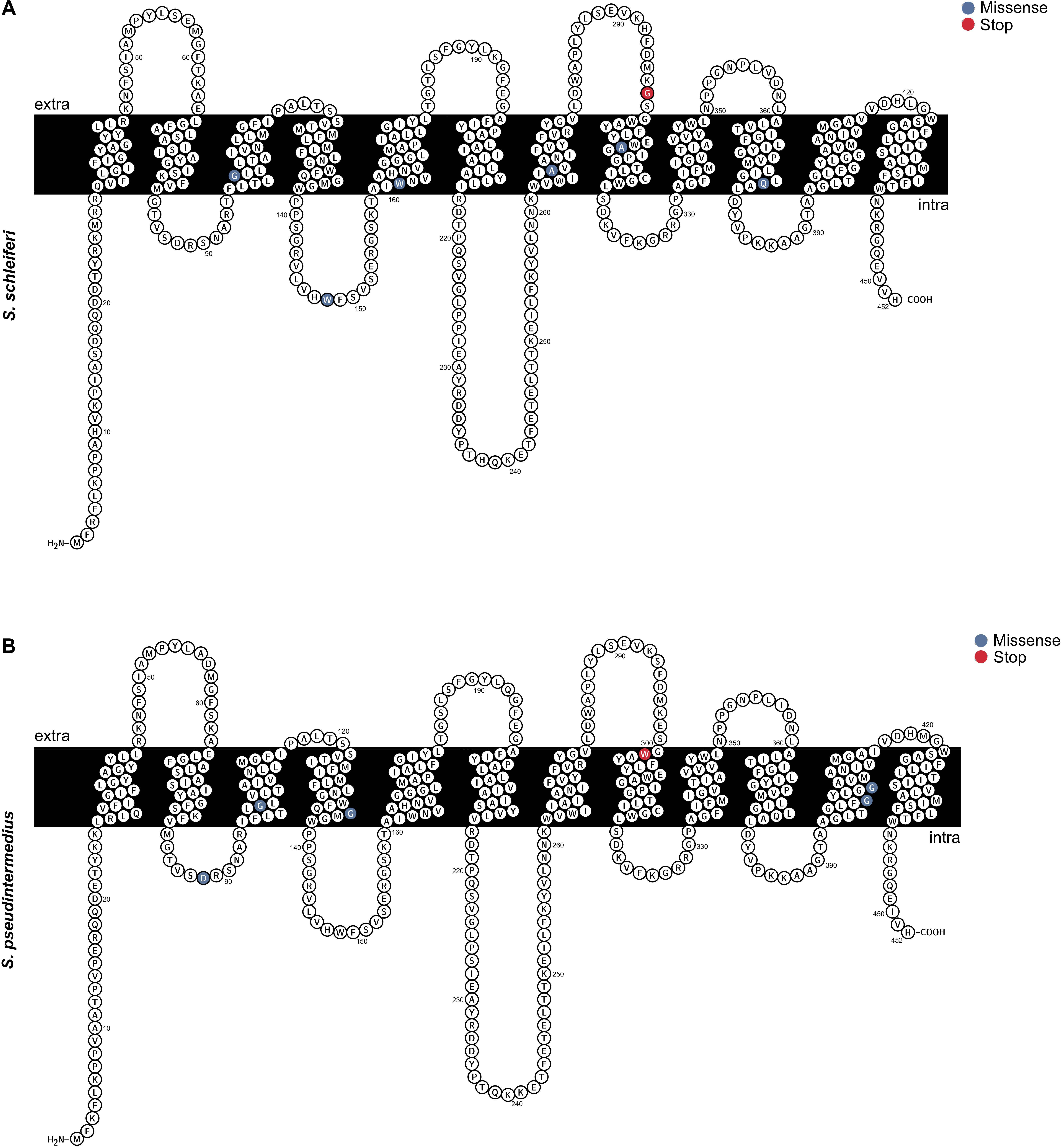

Supplement: S3 Fig — (A) Wild-type amino acid sequences and predicted transmembrane topology of S. schleiferi GlpT. Residues Gly-99, Trp-148, Trp-161, Ala-267, Gly-298, Ala-309, and Gln-379 are indicated in the sequence. Red indicates a stop mutation at the site, while blue indicates a missense mutation. (B) Wild-type amino acid sequences and predicted transmembrane topology of S. pseudintermedius GlpT. Residues Asp-88, Gly-99, Gly-135, Trp-301, Gly-400, and Gly-404 are indicated in the sequence. Red indicates a stop mutation at the site, while blue indicates a missense mutation. Schematic diagrams were prepared with the program Protter(82). (TIFF) [file ppat.1007806.s003.tiff]
