# Supplementary material for: Potent, specific MEPicides for treatment of zoonotic staphylococci
Source: PLoS Pathog. 2020 Jun 4;16(6):e1007806. doi: 10.1371/journal.ppat.1007806 (PMC7297381; doi:10.1371/journal.ppat.1007806)
Supplement: S3 Table — (DOCX) [file ppat.1007806.s006.docx]

**Table S3. FSM MICs, *glpT* alleles, GlpT protein changes, and Polyphen-2 scores for FSM^R^ strains.**

| **Strain** | **FSM MIC (μm)** | ***glpT* change** | **GlpT protein variant** | **Polyphen-2 score*** |
| --- | --- | --- | --- | --- |
| Parental  *S. schleiferi* | 19.8 | N/A | N/A | N/A |
| SSCH3717 | >1500 | N/A | N/A | N/A |
| SSCH8586 | >1500 | G296A | G99E | 1.00 |
| SSCH4455 | >1500 | G892T | G298X | N/A |
| SSCH7376 | 437.5 | G444T | W148C | 1.00 |
| SSCH8774 | >1500 | A1136C | Q379P | 0.967 |
| SSCH3408 | 500 | C800A | A267E | 0.996 |
| SSCH1903 | 718.8 | G295T | G99X | N/A |
| SSCH8400 | 468.8 | G482T | W161L | 0.999 |
| SSCH4494 | 750 | G295T | G99X | N/A |
| SSCH3612 | 687.5 | G296T | G99V | 0.996 |
| SSCH3624 | 687.5 | G296T | G99V | 0.996 |
| SSCH3624 | 562.5 | G925T | A309S | 0.642 |
| Parental  *S. pseudintermedius* | 25.9 | N/A | N/A | N/A |
| SPSE1933 | 875 | G296A | G99E | 1.00 |
| SPSE2648 | 1000 | G296A | G99E | 1.00 |
| SPSE7104 | 1250 | G296A | G99E | 1.00 |
| SPSE6794 | 1250 | G296A | G99E | 1.00 |
| SPSE9765 | 1250 | N/A | N/A | N/A |
| SPSE8628 | 1062.5 | N/A | N/A | N/A |
| SPSE6909 | 687.5 | G1211A | G404D | 1.00 |
| SPSE2227 | 1125 | G404A | G135D | 1.00 |
| SPSE5869 | 937.5 | G404T | G135V | 1.00 |
| SPSE7724 | 1125 | G1199T | G400C | 0.996 |
| SPSE0643 | 1125 | G902A | W301X | N/A |
| SPSE2052 | 1125 | G262T | D88Y | 1.00 |

* = Polyphen-2 scores cannot be calculated for truncation mutations. Polyphen-2 is an algorithm for predicting the probability of deleterious effects of missense mutations. HumDiv scores were used. Whole genome sequencing data is deposited in the NCBI BioProject and Sequence Read Archive databases, PRJNA488092.
