# Supplementary material for: Potent, specific MEPicides for treatment of zoonotic staphylococci
Source: PLoS Pathog. 2020 Jun 4;16(6):e1007806. doi: 10.1371/journal.ppat.1007806 (PMC7297381; doi:10.1371/journal.ppat.1007806)
Supplement: S1 File — (DOCX) [file ppat.1007806.s009.docx]

**Supporting Information**

**Potent, specific MEPicides for treatment of zoonotic staphylococci**

Running Title: MEPicides for zoonotic staph

Rachel L. Edwards^1*^, Isabel Heueck^1^, Soon Goo Lee^2^, Ishaan T. Shah^1^, Andrew J. Jezewski^3^, Justin J. Miller^1^, Marwa O. Mikati^1^, Xu Wang^4^, Robert C. Brothers^4^, Kenneth M. Heidel^4^, Carey-Ann D. Burnham^1,3,5^, Sophie Alvarez^6^, Stephanie A. Fritz^1^, Cynthia S. Dowd^4^, Joseph M. Jez^7^, Audrey R. Odom John^1,3*^

^1^Department of Pediatrics, Washington University School of Medicine, St. Louis, MO, USA; ^2^University of North Carolina-Wilmington, Wilmington, NC, USA; ^3^Department of Molecular Microbiology, Washington University School of Medicine, St. Louis, MO, USA; ^4^Department of Chemistry, George Washington University, Washington, DC, USA; ^5^Department of Pathology and Immunology, Washington University School of Medicine, St. Louis, MO, USA; ^6^Department of Agronomy and Horticulture, University of Nebraska-Lincoln, Lincoln, NE, USA ; ^7^Department of Biology, Washington University, St. Louis, MO, USA.

Present address: Isabel Heueck, Sandoz Deutschland/Hexal AG, Oberhaching, Germany; Xu Wang, Brown University Providence, RI, USA; Soon Goo Lee, University of North Carolina-Wilmington, Wilmington, NC, USA; Robert C. Brothers, Naval Surface Warfare Center, Indian Head, MD, USA.

**Supplementary Methods**

**Specimen collection and processing.** In a study of *Staphylococcal* transmission dynamics in households of children with recent methicillin-resistant *Staphylococcus aureus* infections, culture swabs (Eswab, COPAN, Murrieta, CA and minitip Eswab, Becton Dickinson [BD], Franklin Lakes, NJ) were collected from the dorsal fur and anterior nares/mouth of indoor pet dogs and cats(1)(2). Standard culturing methods were used to assess colonization with *S. aureus*, *S. pseudintermedius*, or *S. schleiferi*. Briefly, tryptic soy broth (TSB) with 6.5% NaCl (BBL, BD) was inoculated with 100 µL eluate from each colonization swab and incubated overnight at 35°C in ambient air, followed by 100 µL inoculation to tryptic soy agar with 5% sheep blood (BBL, BD) and overnight incubation at 35°C. Identification of *S. aureus*, *S. pseudintermedius*, or *S. schleiferi* and antibiotic susceptibility testing for oxacillin (for *S.* pseudintermedius and *S. schleiferi*)/cefoxitin (for *S. aureus*), trimethoprim-sulfamethoxazole, and doxycycline were conducted in accordance with established techniques (CLSI)(1). All isolates recovered from pets were identified using matrix-assisted laser desorption ionization time-of-flight mass spectrometry with the VITEK MS v2.0(3). Isolates were then frozen in TSB with 20% glycerol (BBL, BD) at -80°C.

**Growth inhibition assays for Gram-negative bacteria.** Overnight cultures of *Escherichia coli* (BW25113; Keio Collection), *Klebsiella pneumoniae* (ATCC 43816; gift from David Hunstad), *Burkholderia thailandensis* (gift from Timothy Hagen), and *Salmonella typhimurium* (strain 04-018-0254), *Shigella sonnei*, and *Serratia marcescens* (strain 137.01) (gifts from Phillip Tarr) were diluted 1:200-1:500 in LB media and grown at 37°C until the mid-logarithmic phase (OD_600_ = 0.5 – 0.8). Cultures were diluted in a 96-well plate to 1 x 10^5^ in 150 µL LB media and treated with inhibitors at concentrations ranging from 20 nM to 100 μM. Bacteria were grown at 37°C for 22 h with cyclic shaking at 700 rpm in a FLUOstar Omega microplate reader (BMG Labtech). Growth was assessed over 22 h by measuring the OD_600_ at 20 min increments. The half maximal inhibitory concentration (IC_50_) values were determined during logarithmic growth using GraphPad Prism software. All experiments were performed at least in triplicate and data reported represent the mean ± SD.

***Staphylococcal* genomic DNA isolation.** DNA was isolated as previously described with slight modifications(4). Briefly, 10 mL from an overnight culture was harvested by centrifugation at 7598 x g for 10 min at 10°C. The pellet was washed twice with 10 mL Dulbecco’s phosphate buffered saline (Gibco) and centrifuged at 7598 x g for 15 min at 10°C. Then, 0.5 mL of 10 mM Tris-Hydrochloric acid (HCl) pH 8 and 2.5 mg/mL of lysozyme was added, and the sample was incubated at 37°C for 1-2 h in a water bath. Proteinase-K digestion was performed by adding 1 mL of lysis buffer pH 8 (50 mM Tris, 100 mM EDTA, 1 % SDS) and 1 mg/mL proteinase-K and incubation at 37°C for 1-2 h in a water bath. The genomic DNA was isolated via phenol:chloroform extraction and the DNA precipitated with EtOH. The DNA was resuspended in Tris-EDTA and stored at -20°C.

**References**

1. Clinical and Laboratory Standards Institute. CLSI. Performance Standards for Antimicrobial Susceptibility Testing. CLSI Supplement M100. 28th ed. Wayne, PA; 2018.

2. Fritz SA, Hogan PG, Singh LN, Thompson RM, Wallace MA, Whitney K, et al. Contamination of environmental surfaces with *Staphylococcus aureus* in households with children infected with methicillin-resistant *S. aureus*. JAMA Pediatr. 2014;168(11):1030.

3. Rychert J, Burnham C-AD, Bythrow M, Garner OB, Ginocchio CC, Jennemann R, et al. Multicenter evaluation of the Vitek MS matrix-assisted laser desorption ionization-time of flight mass spectrometry system for identification of Gram-positive aerobic bacteria. J Clin Microbiol. 2013;51(7):2225–31.

4. Kumar R, Yadav BR, Dev K, Singh RS. Protocol Online: a simple protocol for DNA extraction from *Staphylococcus aureus*. Protocol Online. 2008.

5. Omasits U, Ahrens CH, Müller S, Wollscheid B. Protter: interactive protein feature visualization and integration with experimental proteomic data. Bioinformatics. 2014;30(6):884–6.
